# MUC2 mucin deficiency alters inflammatory and metabolic pathways in the mouse intestinal mucosa

**DOI:** 10.18632/oncotarget.16886

**Published:** 2017-04-06

**Authors:** Selamawit Tadesse, Georgia Corner, Elena Dhima, Michele Houston, Chandan Guha, Leonard Augenlicht, Anna Velcich

**Affiliations:** ^1^ Department of Medicine, Albert Einstein College of Medicine/Albert Einstein Cancer Center, NY, USA; ^2^ Department of Radiation Oncology, Albert Einstein College of Medicine/Albert Einstein Cancer Center, NY, USA; ^3^ Current Address: Flow Cytometer Resource Center, The Rockefeller University, NY, USA

**Keywords:** MUC2, mucin, inflammation, metabolism, tumorigenesis

## Abstract

The mucus layer in the intestine affects several aspects of intestinal biology, encompassing physical, chemical protection, immunomodulation and growth, thus contributing to homeostasis. Mice with genetic inactivation of the *Muc2* gene, encoding the MUC2 mucin, the major protein component of mucus, exhibit altered intestinal homeostasis, which is strictly dependent on the habitat, likely due to differing complements of intestinal microbes. Our previous work established that *Muc2* deficiency was linked to low chronic inflammation resulting in tumor development in the small, large intestine including the rectum. Here, we report that inactivation of Muc2 alters metabolic pathways in the normal appearing mucosa of *Muc2^−/−^* mice. Comparative analysis of gene expression profiling of isolated intestinal epithelial cells (IECs) and the entire intestinal mucosa, encompassing IECs, immune and stromal cells underscored that more than 50% of the changes were common to both sets of data, suggesting that most alterations were IEC-specific. IEC-specific expression data highlighted perturbation of lipid absorption, processing and catabolism linked to altered Pparα signaling in IECs. Concomitantly, alterations of glucose metabolism induced expression of genes linked to *de novo* lipogenesis, a characteristic of tumor cells. Importantly, gene expression alterations characterizing *Muc2^−/−^* IECs are similar to those observed when analyzing the gene expression signature of IECs along the crypt-villus axis in WT B6 mice, suggesting that *Muc2^−/−^* IECs display a crypt-like gene expression signature. Thus, our data strongly suggest that decreased lipid metabolism, and alterations in glucose utilization characterize the crypt proliferative compartment, and may represent a molecular signature of pre-neoplastic lesions.

## INTRODUCTION

A unique set of rules has evolved to control the potential constant stimulation of the intestinal immune system that could arise from the intestinal microbiota. This in part takes the form of a conversation between the epithelial cells of the intestinal mucosa and the gut microbiota that keeps inflammation in check and plays a fundamental role in maintaining intestinal mucosal homeostasis.

A key component modulating the conversation between the mucosa and the vast population of bacteria that inhabit the large intestine is the thick mucus layer that provides a physical separation between the epithelial cell monolayer and the luminal contents. This mucus layer also provides a chemical and biochemical barrier that supports the structure of the mucus gel, as well as the ability of the gel to concentrate biological factors secreted by mucosal epithelial cells (reviewed in [1]). Mucus is also a source of nutrients for the intestinal microbiota, underscoring a complex role of the mucus layer. In addition, in the small intestine, the mucus, and goblet cells, which secrete the mucin MUC2, have an active role in regulating mucosal immunity [2, 3]. Thus, compromising the integrity of the mucus barrier has profound effects on the functioning and homeostasis of the intestinal mucosa.

We reported that targeted inactivation of the *Muc2* gene in the mouse, which encodes the major intestinal mucin MUC2, eliminates the mucus barrier resulting in physical contact between intestinal bacteria and the mucosa. This generates a chronic, low-level inflammatory response, and eventual tumor development throughout the small and large intestine, and the rectum [4]. The importance of this subtle inflammatory response in *Muc2*^−/−^ mice was also evident from exacerbation of the tumor phenotype in double mutant *Muc2*^−/−^; *Apc*^1638N/+^ or *Muc2*^−/−^; *Apc*^Min/+^ mice [5]. *Muc2* deficiency not only accelerated the kinetics of tumor formation and increased tumor number initiated by loss of Apc function, but also shifted tumor location, with an increased tumor load in the colon. Importantly, these effects were *Muc2* dosage dependent, as *Muc2* haploinsufficiency caused similar, but more modest, changes. The tumor phenotype of the double mutant *Muc2/Apc* mice recapitulated the phenotype of *Apc* mutant mice challenged to mount an inflammatory response [6, 7], strongly suggesting that the inflammatory stimulus due to loss of the mucus barrier was a key event in exacerbating initiation of tumor development caused by loss of Apc function.

Because the mucus barrier plays such a fundamental role in intestinal functions and homeostasis, it is likely that absence of the barrier profoundly alters homeostasis in the intestinal mucosa. In this study, we used gene expression profiling of both isolated epithelial cells of the intestinal mucosa, as well as of mucosal tissue that contains epithelial as well as non-epithelial cells, to determine extent and nature of the perturbations of the mucosa in *Muc2*^−/−^ compared to wild-type mice. The expression data and follow-up experiments demonstrated two major classes of alteration in *Muc2*^−/−^ mucosa: first, altered immune cell functions and DNA repair as a sequela to damage of the mucosa; second, decreased ability of intestinal cells to absorb, process and secrete lipids back into circulation, and an impairment of IECs in catabolizing fatty acids, coupled with an increase in the ability of epithelial cells to take up glucose and alterations in its utilization.

## RESULTS

To dissect alterations in the intestine of *Muc2*^−/−^ mice, we compared the expression profile of isolated intestinal epithelial cells (IECs) from 3 month old *Muc2*^−/−^ and control *Muc2*+/+ mice. We also compared the expression profile of the entire mucosal cell population of *Muc2*^−/−^ and *Muc2*^+/+^ mice, which includes intestinal epithelial cells as well as stromal and immune cells. We focused on the duodenal cell population, the site displaying the highest frequency of tumor development, as we reported earlier [4].

We first identified changes that satisfied 2 criteria: a magnitude of difference > 1.5 fold coupled with a *p* value of ≤ 0.05 (Student's *t*-test). Of 260 sequences meeting these criteria in the total heterogeneous cell population of the *Muc2*^−/−^ intestinal mucosa, 162 were also detected among the changes in the purified epithelial cells. Thus, approximately 100 sequences most likely represent alterations in the stroma and immune cell compartment.

**Figure 1:**
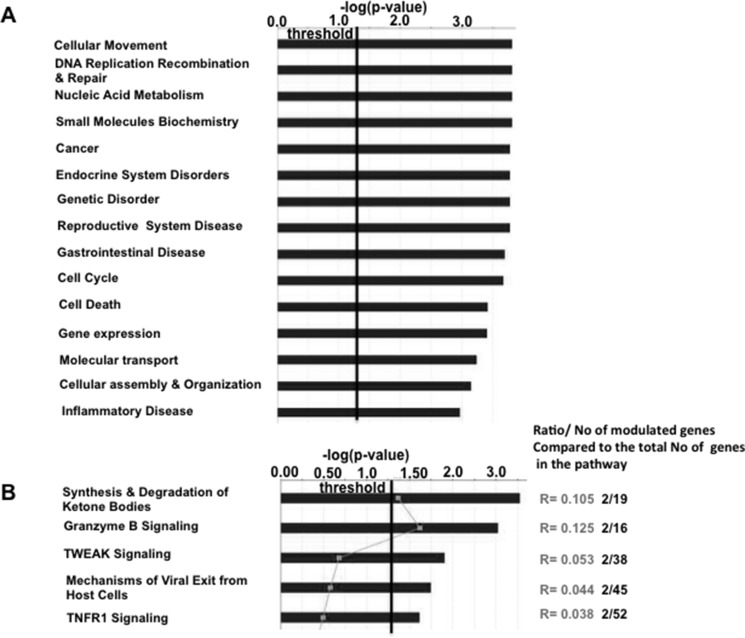
Modest alterations are detected in the intestinal non- epithelial cell compartment of *Muc2*^−/−^ mice Ingenuity pathway analysis (IPA) was used to identified gene expression changes that affect biological functions (**A**), and specific cellular pathways (**B**).

Bioinformatic analysis of these data (IPA, Ingenuity System Software) identified 15 different biological functions enriched in the altered expression profile of the *Muc2*^−/−^ stromal cell population (Figure 1A). However, these changes could be aligned to only 5 discrete pathways (Figure 1B). It is notable that for these 5 pathways, the number of genes which showed detectable alterations in expression was limited to 5–10% of the genes in each pathway, indicating a specific response of the mucosa to the loss of the mucus barrier (Figure 1B).

**Figure 2:**
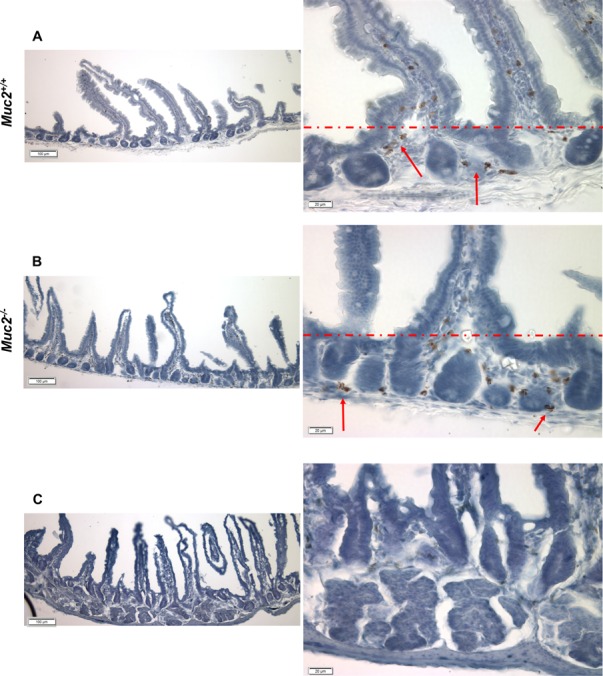
Detection of ROS producing cells in the stromal compartment of *Muc2*^+/+^ and *Muc2*^−/−^ mice (**A**–**C**) show representative micrographs of frozen sections of flat mucosa of small intestine from 3 month old *Muc2*^+/+^(A) and *Muc2*^−/−^ (B) mice, respectively, incubated *in vitro* with DAB, as described in Material and Methods. Red arrows indicate cell positivity due to DAB polymerization in the presence of cell-generated H_2_O_2_. (C) shows loss of signal, and thus specificity of the reaction, upon addition of catalase to the reaction buffer. The red dotted line separates the intravillus area from the pericrypt area. Magnification bars are shown.

**Figure 3:**
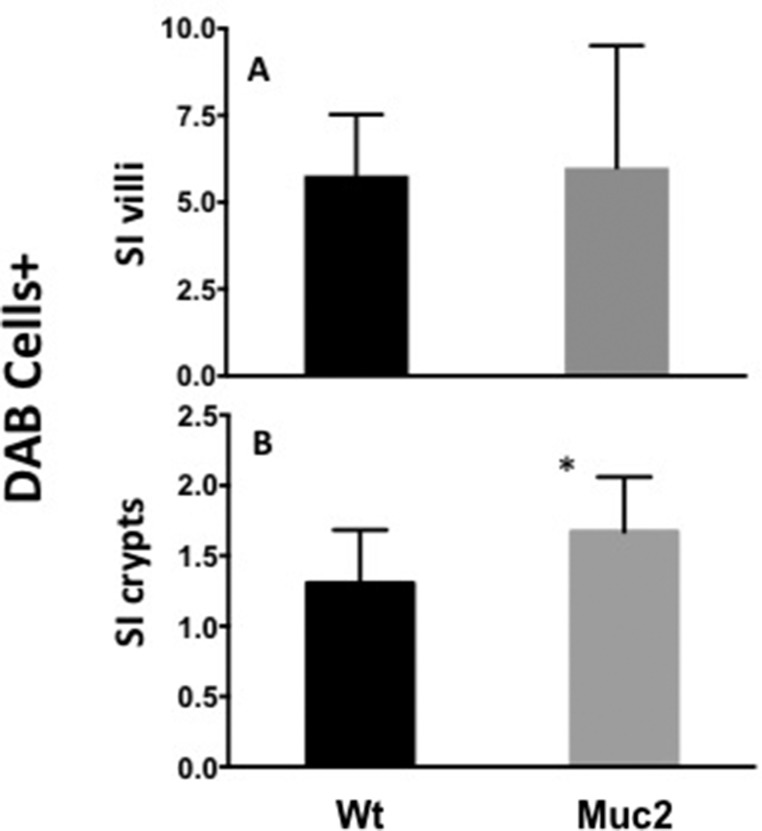
Increased number of ROS positive cells in *Muc2*^−/−^ mice Quantification of ROS producing cells in sections from *Muc2*^+/+^ and *Muc2*^−/−^ mice. The number of DAB+ cells was determined in tissue sections incubated with DAB, as shown in Figure 2: (**A**) in the intravillus stroma; (**B**) in the stroma surrounding the crypts of the small intestine (SI) of 3 month old mice of the indicated genotypes. A minimum of 100 villi/crypts in well oriented sections were analyzed/ mouse, 3 mice/ genotype. (error bars, s.d.; Student's *t* test, *p < 0.05)

The altered biological functions identified in the *Muc2*^−/−^ intestine involved genes associated with cell movement (Figure 1A), including several genes responsible for migration of immune cells. Specifically, this included genes involved in chemotaxis and migration of neutrophils and macrophages, exemplified by upregulation of C-C motif chemokine ligand 8 (*CCl8*), Serum amyloid A1 (*Saa1*), Neuromedin U (*Nmu*), and Endothelin 3 (*Edn3*). These data, along with upregulation of Stomatin, a component of neutrophil granules and phagosomes, prompted investigation of whether there was an increase in phagocytes in the intestine of *Muc2* mice. Phagocytes respond to stimuli with an oxidative burst characterized by ROS production, which can be visualized as an insoluble brown precipitate formed by oxidized and polymerized DAB in the presence of endogenously produced H_2_O_2_ and activated peroxidase [8]. As shown in Figure 2, there were many more DAB positive cells in the small intestine (compare 2A to 2B) of the *Muc2*^−/−^ mouse. In H&E stained serial sections, these cells were identified as PMNs (data not shown). Loss of DAB staining by the addition of catalase to the incubation buffer, an enzyme that metabolizes H_2_O_2_, demonstrated specificity of the reaction (Figure 2C). These data were confirmed by fluorescent detection of ROS producing cells using an oxidized DCF probe in the small intestine: Supplementary Figure 1 shows increased number of DCF positive – ie, green fluorescent-cells - in the stroma surrounding the crypts of *Muc2*^−/−^ small intestine (S1B) compared to wild-type mice (S1A). Importantly, quantitative analysis of DAB^+^ cells (Figure 3) demonstrated that DAB^+^ cells were similarly represented in the intravillus stromal compartment of *Muc2*^−/−^ and wild-type mice (Figure 3A), with a significant increase in the number of DAB^+^ cells in the pericrypt stroma (Figure 3B) of the duodenum of *Muc2*^−/−^ mice.

**Figure 4:**
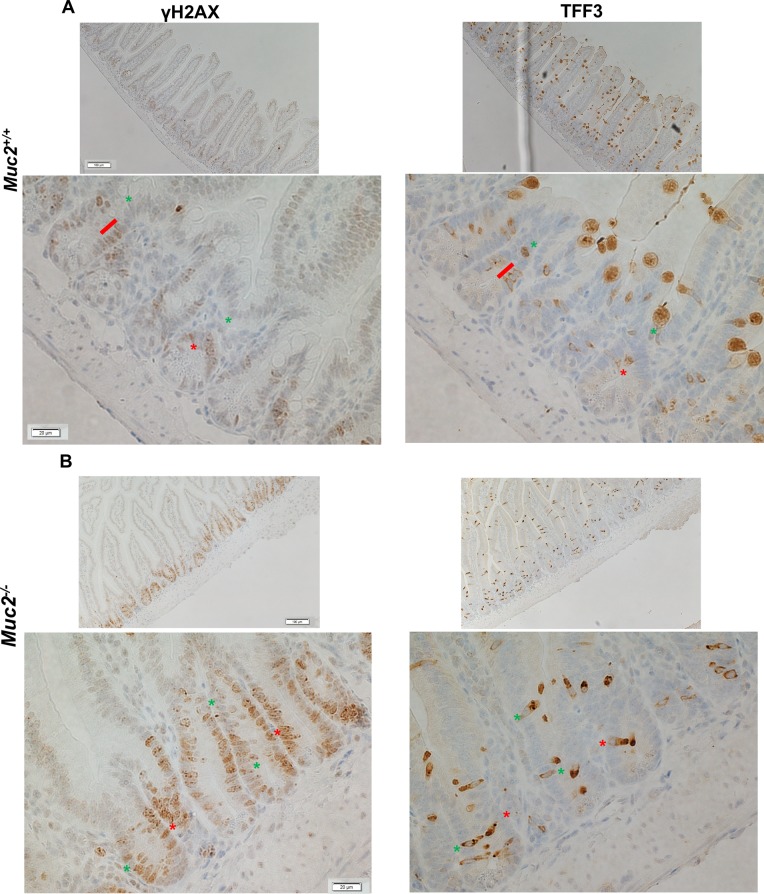
Increased number of γH2AX positive cells in *Muc2*^−/−^ crypts Immunohistochemical detection of γH2AX (left column), a marker for DNA damage, and TFF3 (right column), a marker of goblet cells in serial sections of SI from 3 month old mice of the indicated phenotypes, (**A**) and (**B**) for *Muc2*^+/+^ and *Muc2*^−/−^, respectively. In A and B red lines and asterisks identify γH2AX-positive goblet cells, expressing TFF3. Green asterisks pinpoint at γH2AX-positive cells that do not express TFF3.

**Figure 5:**
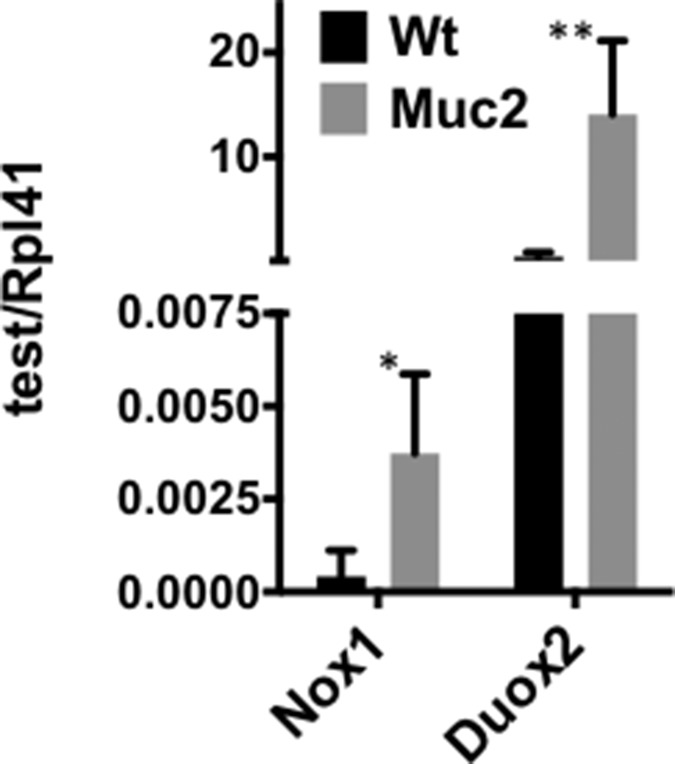
Elevated expression of genes encoding ROS generating enzymes in *Muc2*^−/−^ IECs qRT-PCR was used to determine the levels of mRNAs for Nox1, a NADPH oxidase subunit at the cell membrane, and Duox2, a dual oxidase in SI-IECs from WT and *Muc2*^−/−^ mice.

To determine whether the increased ROS contribute to DNA damage in IECs, the accumulation of γH2AX, a marker of DNA damage, was determined in IECs from the small intestine. As shown in Figure 4A–4B, and quantified in Supplementary Figure 2, there was a distinct increased number of γH2AX positive IECs, that also showed greater staining intensity, in the small intestine of *Muc2*^−/−^ (S2B) compared to wild-type mice (S2A). Further, γH2AX positive cells were localized in the crypts, where the increased number of ROS positive phagocytes was detected (Figures 2 and 3). Further, IECs may also be an additional source of ROS in response to increased exposure to the intestinal bacteria, as we previously reported in the intestine of the Muc2 mouse [9]. Indeed, Figure 5 shows elevated expression of NAPDH oxidase 1 (*Nox1*) and Dual oxidase 2 (*Duox2*), ROS generating enzymes that are modulated in response to bacteria [10–13], as determined by RTqPCR. Of note, the expression of these two genes was not significantly changed in the array results. These data suggest that elevated ROS production by the infiltrating phagocytes, and IECs, promote genomic instability in epithelial cells, a potential driver of tumor development, either directly and/or by altering the microenvironment. However, it cannot be ruled out that the increased number of γH2AX positive cells in mutant mice may be linked to the hyperproliferation documented in *Muc2*^−/−^ crypts [4].

It was recently reported that ROS are required for goblet cell functions, including mucin secretion [14]. Therefore, we ascertained whether γH2AX localized preferentially to goblet cells in the crypts of *Muc2*^−/−^ mice. Serial sections were stained for γH2AX and TFF3, the latter a goblet specific marker that we showed is equivalently expressed in WT and *Muc2*^−/−^ intestine [4], and serves as a surrogate marker for goblet cells when they are devoid of MUC2. Figure 4A and 4B (left panel γH2AX, right panel TFF3, respectively), show that the great majority of γH2AX positive cells in WT and *Muc2*^−/−^ crypts of the small intestine were not goblet cells by the criteria of absence of TFF3 expression.

Muc2, the fundamental component of the mucus barrier, is expressed and secreted by goblet cells in both the small and large intestine. Not surprisingly, the inactivation of the *Muc2* gene leads to the absence of recognizable goblet cells whose distinctive morphology is due to the storage of mucin [4]. The histological changes in the mucosa of *Muc2* mice extend beyond this, however, to a general narrowing of crypt-villus architecture coupled with an acceleration of cell migration along the crypt-luminal axis [4]. Consistent with these perturbations in structure, there were also robust changes in gene expression in purified epithelial cells of the mucosa. This was analyzed utilizing the IPA Ingenuity system applied again to sequences that were altered ≥ 1.5 fold in expression coupled with a *p* ≤ 0.05 in isolated IECs of the *Muc2*^−/−^ compared to wild-type mice. The most altered biological networks were linked to drug and lipid metabolism, small molecule biochemistry, and molecular transport (data not shown), in addition to other metabolic processes altered in the intestine of *Muc2*^−/−^ mice relative to wild-type mice. This is particularly relevant, as recent studies have identified the gut microbiota, and their combined genetic potential, the microbiome, as an important environmental determinant in influencing host energy metabolism [15].

**Figure 6:**
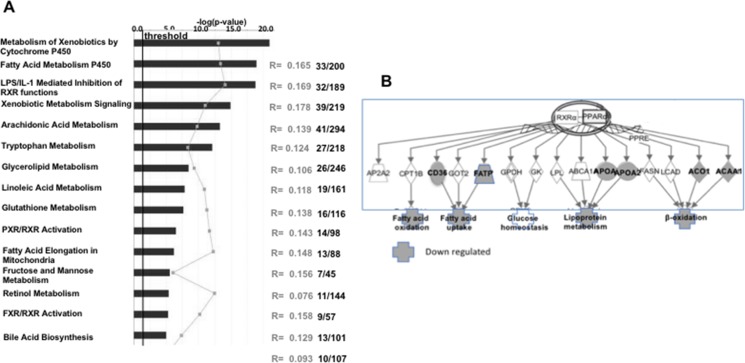
Major alterations are detected in SI-IECs of *Muc2*^−/−^ mice: PPARα downregulation characterizes altered lipid metabolism in *Muc2*^−/−^ IECs Ingenuity pathway analysis (IPA) was used to identified gene expression changes that affect specific cellular pathways (**A**). In (**B**), IPA bioinformatics was used for the merger of the top 2 networks. The analysis confirms major changes in several aspects of lipid metabolism, and identifies PPARa as a prominent regulator of these processes. Symbols in grey indicate genes, and affected pathways, that are downregulated in *Muc2*^−/−^ IECs.

There were 36 canonical pathways that were significantly modulated. The most prominently modulated, both by fold change and *p* value, were xenobiotic and fatty acid metabolism (Figure 6A). The merger of the top 2 networks pinpoints major changes in several aspects of lipid metabolism, and identifies PPARa as a potential common regulator of those processes (Figure 6B).

**Figure 7:**
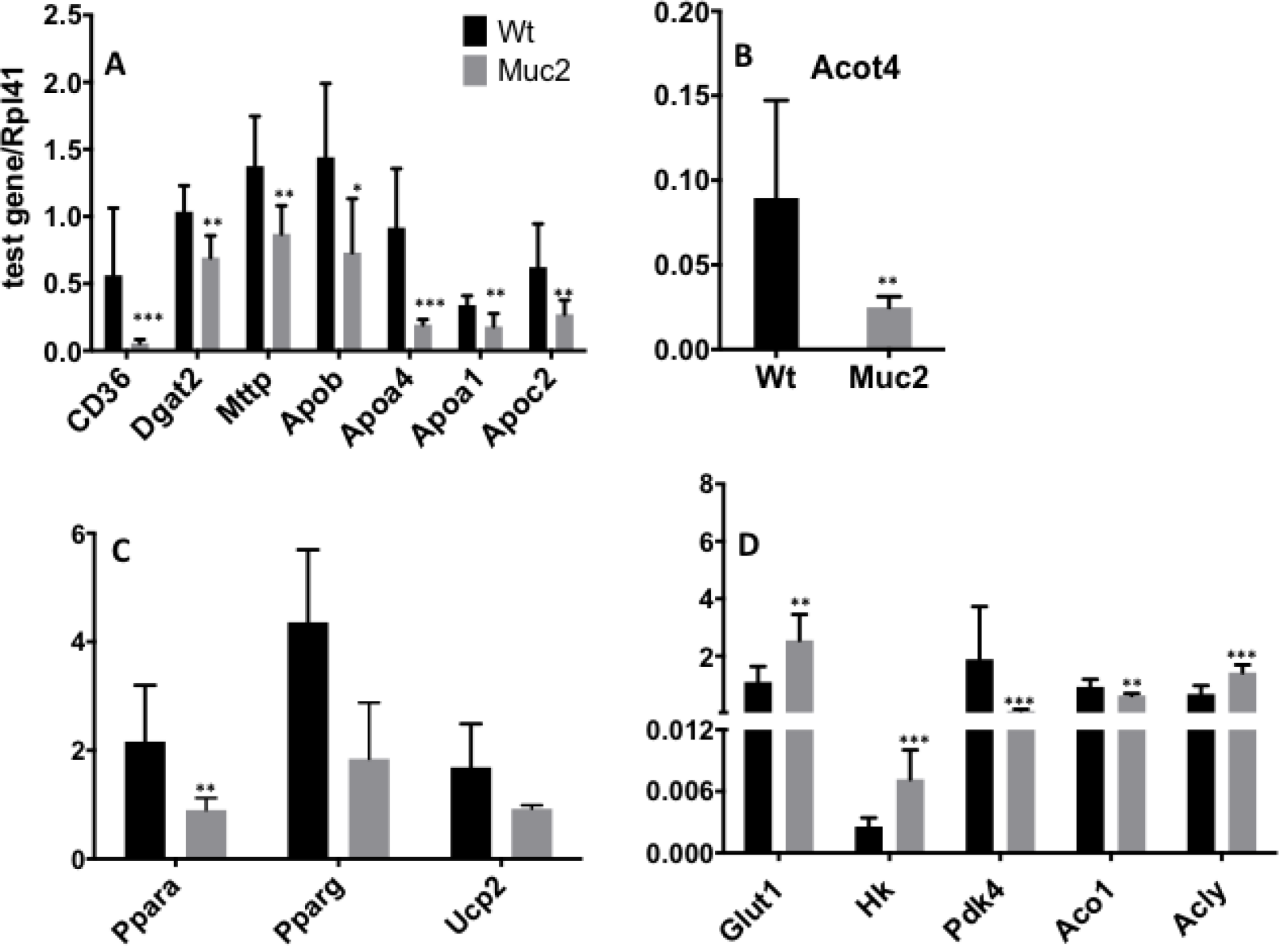
Muc2 deficiency alters the expression of genes associated with lipid metabolism Validation by quantitative real-time polymerase chain reaction (qRT-PCR) of the levels of mRNAs encoded by each of the indicated genes involved in the regulation of lipid and glucose metabolism. Total RNA was isolated from purified SI-IECs from 3 month old WT and *Muc2*^−/−^ mice.

Alterations of lipid, and specifically fatty acid metabolism, can be broadly subdivided into two main categories: those affecting the uptake and distribution of fatty acids in the organism, and those altering their utilization by intestinal epithelial cells. Our previous work had shown that fatty acid binding proteins (Fabps), facilitating the transport of fatty acids to the ER, were down-regulated in *Muc2*^−/−^ IECs [5]. We now extend these observations and show in Table 1 that the expression of genes involved in sequential steps of lipid metabolism and associated functions was significantly decreased in the array data. qRT-PCR validation confirmed that this process was severely perturbed in *Muc2*^−/−^ IECs: among the down-regulated genes were those encoding fatty acid transporters CD36 and Solute carrier family 27 member 4/Fatty acid transporter protein 4 (Scl27a4/Fatp4), the ER associated enzymes Diacylglycerol O-Acyltransferase 2 (Dgat2) and Microsomal Triglyceride Transfer Protein (Mttp), and those encoding mediators of the final assembly of chylomicrons, including Apolipoprotein B (ApoB), ApoAIV, ApoA1 and ApocII (Figure 7A).

**Table 1:** Genes involved in fatty acid metabolism

		Log2	Direct targets		
UniGene	Gene Symbol	Differences	*t* Test	Ppar-α*	Function
Mm.330113	Slc27a4/Fatp4	−1.2597	0.0454		**Fatty Acid uptake and Chilomicrone formation**
Mm.18628	CD36	−2.4442	0.0063	√	
Mm.2941	Mttp	−0.9460	0.0036	√	
Mm.208030	Mogat2	−0.8522	0.0219		
Mm.22633	Dgat1	−0.7512	0.0633	√	
Mm.477728	Dgat2	−1.5784	0.0010		
Mm.4533	Apoa4	−1.8770	0.0139	√	
Mm.26743	Apoa1	−0.9414	2.18E-05	√	
Mm.221239	Apob	−1.2659	0.0342		
Mm.477720	Apoc2	−1.6412	0.0047		
					**Fatty Acid Degradation**
Mm.260164	Hadh	−0.5912	0.0208	√	Mitochondria β-oxidation
Mm.379011	Scp2	−0.8594	0.0003		Mitochondria β-oxidation
Mm.210323	Acsl1	−0.7173	0.0429	√	Mitochondria β-oxidation
Mm.245724	Acaa2	−0.7438	0.0095	√	Mitochondria β-oxidation
Mm.292056	Acsl5	−1.0342	0.0145	√	Fatty_Acid_Degradation
Mm.20396	Crat	−0.8403	0.0004	√	Fatty_Acid_Degradation
Mm.246682	Gyk	−1.2075	0.0082	√	Fatty_Acid_Degradation
Mm.42253	Slc22a5/Octn2	−0.8247	0.0138	√	carnitine uptake
Mm.399042	Abcd3	−0.7621	0.0275		VLFA/peroxisomal β-oxidation
Mm.290044	Slc27a2/Fatp2	−1.1491	0.0672	√	peroxisomal β-oxidation
Mm.205266	Acaa1a /1b	−0.9283	0.0014		peroxisomal β-oxidation
Mm.475660	Acot4	−1.5366	0.0063		peroxisomal β-oxidation
Mm.439978	Cyp4a10//Cyp4a31	−3.1568	0.0005	√	ɷ-oxidation
Mm.1840	Cyp4b1	−1.9094	0.0163		ɷ-oxidation
Mm.30504	Cyp4f16	−0.9681	0.0005		ɷ-oxidation

^*^References [16, 17]

**Table 2:** Modulation of genes of Fatty Acid metabolism along the crypt/villus axis

	Muc2/Wt	F10/F1	
Gene Symbol	Differences log2		*t* Test
Slc27a4/Fatp4	−1.2597	−2.5085	0.0000
CD36	−2.4442	−1.5082	0.0209
Mttp	−0.9460	−1.2746	0.0001
Mogat2	−0.8522		
Dgat1	−0.7512	−0.8729	0.3972
Dgat2	−1.5784	−2.8222	0.0000
Apoa4	−1.8770	−1.6981	0.0002
Apoa1	−0.9414	−0.6254	0.0002
Apob	−1.2659	−3.3390	0.0004
Apoc2	−1.6412	−2.9857	0.0022
Hadh	−0.5912	−1.5576	0.0143
Scp2	−0.8594	−1.2758	0.1873
Acsl1	−0.7173	−0.7345	0.0965
Acaa2	−0.7438		
Acsl5	−1.0342	−1.3321	0.0014
Crat	−0.8403		
Gyk	−1.2075	−2.9896	0.0000
Slc22a5/Octn2	−0.8247		
Abcd3	−0.7621	−0.8979	0.0067
Slc27a2/Fatp2	−1.1491	0.0511	0.8748
Acaa1a /1b	−0.9283	−1.4126	0.0013
Acot4	−1.5366	−0.6090	0.0487
Cyp4a10//Cyp4a31	−3.1568		
Cyp4b1	−1.9094	−2.5144	0.0002
Cyp4f16	−0.9681	−1.5814	0.0086

F10: crypt cell fraction. F1: villus cell fraction [29].

In *Muc2*^−/−^ IECs, perturbation of lipid metabolism also extended to the catabolism of fatty acids (Table 1). There was reduced expression of ATP binding cassette subfamily D3 (*Abcd3*), encoding a transporter for VLFA in peroxisomes and Fatp2/Slc27a2, a transporter required for activation of fatty acids and subsequent oxidation in peroxisomes, as well as Acyl-CoA Thioestherase 4 (*Acot4*), confirmed by qRT-PCR in Figure 7B, and *Acaa1*, the gene encoding 3-ketoacyl-CoA thiolase B which catalyzes the final step of peroxisomal β-oxidation.

As pointed out in Figure 6B, many of the genes that are modulated by *Muc2* deficiency and that can affect lipid metabolism converge on the major central node represented by Peroxisome Proliferator Activated Receptor α (Pparα) (Figure 6B), and many are direct targets of Pparα (Table 1) [16, 17]. Figure 7C clearly demonstrates that *Ppar*α expression is indeed significantly repressed in *Muc2*^−/−^ IECs.

Pparα regulates key classI/II genes that orchestrate the xenobiotic and oxidative stress response. Consistent with this, our prior work documented that both classes of genes were significantly repressed in *Muc2* ^−/−^ IECs [5]. Further, since there is an inverse relationship between Pparα activation and the down-regulation of genes involved in glucose transport, we investigated whether decreased Pparα expression would be accompanied by increased expression of genes involved in glucose metabolism. Indeed, as shown in Figure 7D, there was significantly increased expression of the genes encoding the glucose transporters *Glut1/Slc2a1*, as well as of *Hk,* which encodes the hexokinase that catalyzes glucose phosphorylation, the first step in glycolysis. Altered glucose utilization has also been linked to modulation of glucose utilization via induction of pyruvate dehydrogenase kinase isoform 4 (*Pdk4*), a Pparα target gene [18]. Accordingly, *Pdk4*, whose product inhibits pyruvate dehydrogenase, which catalyzes the metabolism of pyruvate to Acetyl-CoA, was down-regulated, implying increased pyruvate utilization for acetyl-CoA production in the mitochondria, and promoting an alternative pathway for de novo lipogenesis [19]. This is characteristic of proliferating cells [20], and we have reported elevated proliferation in the intestine of *Muc2*^−/−^ mice. In *Muc2* IECs, the data document decreased expression of Aconitase 1 (*Aco1*), and concomitant elevation of *Acly*, thus, increasing ATP-citrate-lyase (ACL)-dependent production of acetyl-CoA important for the proliferation of tumor cells and lipogenesis [21] (Figure 7D). In addition, the uncouple protein 2 (*Ucp2*), also a target of Pparα activation and downregulated in proliferating cells [22], is down-modulated in *Muc2*^−/−^ IECs (Figure 7C), further supporting the interpretation that the *Muc2* deficient mucosa is characterized by early metabolic alterations promoting anaplerotic reactions for biosynthetic processes required for proliferation.

**Figure 8:**
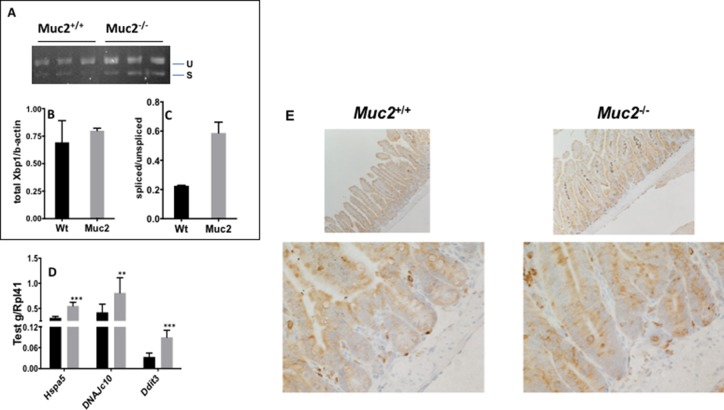
The endoplasmic reticulum (ER) stress response induced in *Muc2*^−/−^ intestinal epithelial cells The stress response was investigated in the SI-IECs of *Muc2*^−/−^ mice by analyzing the relative levels of unspliced *Xbp1*(u) and spliced *Xbp1* (s) mRNA. (**A**) shows the gel analysis of PCR reactions, detecting unspliced and spliced forms of *Xbp1* in purified SI-IECs of mice of the indicated genotypes. (**B**) and (**C**) show quantification of the bands in A for total *Xbp1* mRNA relative to β*-actin* (B) and the relative levels of spliced versus unspliced forms of Xbp1 (C). (**D**) The level of expression of the indicated ER stress responsive genes was determined by qRT-PCR in WT and *Muc2*^−/−^ SI-IECs. (**E**) shows immunohistochemical detection of Grp78 in the small intestine of WT and *Muc2* ^−/−^ mice.

The endoplasmic reticulum (ER) is a prominent site for lipid metabolism, alteration of lipid metabolic pathways, and pathways involved in the detoxification and oxidative stress responses. These are all altered in *Muc2*^−/−^ mice [5], and can induce a stress response in the endoplasmic reticulum termed the “unfolded protein response” (UPR) [23]. Although the *Muc2*^−/−^ IECs did not exhibit a significant elevation of total X-box binding protein 1 (*Xbp1*) expression, we observed a prominent increase of the ratio of spliced (active) versus unspliced *Xbp1* mRNA in *Muc2* IECs (Figure 8A–8C) suggesting that these IECs are under ER stress. Although this altered splicing pattern was not significant, it is consistent with significantly increased levels of Heat Shock Protein family member 5 (*grp78/hspa5/BiP*), an Xbp1 target, and we confirmed upregulation of Grp78 in the crypts of *Muc2* compared to WT mice by immunostaining (Figure 8E). There was also increased expression of Heat Shock Protein (hsp40) family C10 (*DnaJc10*), a stress response gene that encodes a protein disulfide isomerase (PDI) of the ER as well as DNA Damage Inducible Transcript 3 (*Ddit3/Gadd153*) that is also involved in DNA damage response (Figure 8D).

**Figure 9:**
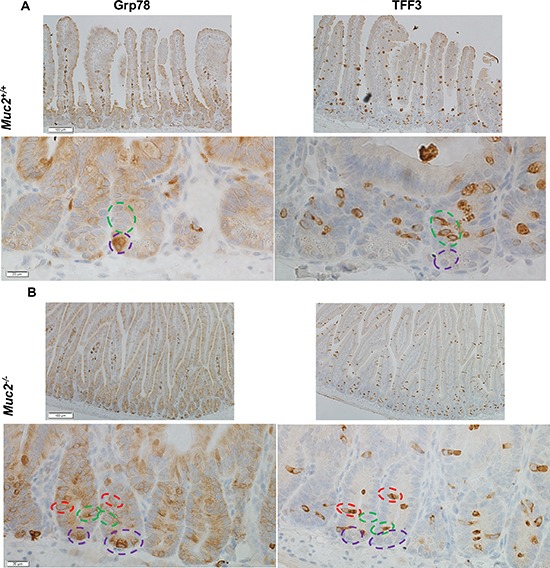
The expression of Grp78 does not preferentially localize to goblet cells Immunohistochemical detection of Grp78, a marker of the ER stress response, left panels, and TFF3, a goblet cell specific marker, right panel, in serial sections of SI of 3 month old mice of the indicated genotype (**A**, WT, and **B**, Muc2). Green dotted circles indicate goblet cells positive for TFF3, but negative for Grp78. Purple dotted circles identify Grp78 positive Paneth cells.

Given the interplay between ROS, ER stress response, and goblet cell functions, we pursued whether the increased Grp78 expression was targeted to goblet cells. Immunohistochemical staining for Grp78 and TFF3 was done on serial sections. Figure 9A and 9B show that in *Muc2*^−/−^ crypts there were occasional goblet cells, identified by positivity for TFF3, also positive for Grp78, yet the great majority of goblet cells were negative for Grp78 both in *Muc2* and WT crypts. However, we observed that the fraction of Grp78 positive Paneth cells was greater in *Muc2* compared to WT mice, in the absence of changes in the absolute number of Paneth cells in mutant and WT mice [4, 5]. These data are consistent with the increased expression of Paneth cell-specific peptides with bacteriocidal and bacteriostatic activity that we and others have previously reported in *Muc2^−/−^* mice [5, 24, 25]

## DISCUSSION

Expression profiling of both isolated epithelial cells and total cells of the intestinal mucosa of *Muc2*^−/−^ mice demonstrated that elimination of the mucus barrier generates significant functional shifts of the intestinal mucosa. These changes encompass alterations in immune cells due to the impact of MUC2 in modulating immune stimulation. This results in a low-level chronic inflammatory response that we documented in the intestine [5]. In addition, we show here that in the intestinal epithelial cells there are major changes in pathways of intermediary metabolism, encompassing altered lipid metabolism, increased utilization of glucose, and ER stress reflected in an unfolded protein response.

These changes are all likely linked to the function of Muc2 as the major component of the mucus barrier, which, when compromised, can alter the intestinal microbiota and the interactions of these bacteria with the mucosa. This is further complicated by the qualitative and quantitative alterations in the composition of the microbiota of *Muc2*^−/−^ mice [24]. This complex interaction with the microbiota in *Muc2*^−/−^ mice is also demonstrated by the rapid death of the mice upon treatment with antibiotic, while in contrast, *Muc2*^+/+^ mice tolerate antibiotic treatment very well (not shown). Alterations of microbiota-epithelial cell interactions are indeed indicated by the data here showing that there is increased number of ROS producing phagocytes in the stroma surrounding intestinal crypts, and upregulation of ROS producing enzymes expressed by IECs contributing to altered homeostasis. Increased ROS production in *Muc2*^−/−^ intestine is indicative of the pathophysiology of the *Muc2*^−/−^ mouse intestine. ROS can exert either negative or positive effects on histopathology depending on the intensity and duration of the oxidative insult. The negative effects of ROS are evident in the link between chronic inflammation, ROS production and cancer. It has been postulated that increased genomic mutation rate is mechanistically linked to tumor development associated with inflammation, which generates oxidative stress [26, 27]. In contrast, ROS, in particular H_2_O_2,_ can also be beneficial through induction of repair processes [28, 29]. Further, in the intestine, commensal bacteria, specifically Lactobacilli that are elevated in the intestine of *Muc2*^−/−^ mice [24], promote ROS production by *Nox1* in enterocytes and modulate epithelial cell proliferation and migration [10, 11], processes that we have previously documented to be increased in the flat mucosa of the *Muc2*^−/−^ mice [4], and further supported by the upregulation of *Nox1* and *Duox*, an additional oxidase involved in antibacterial responses [12, 13], in *Muc2*^−/−^ IECs (Figure 5). Thus, although there is a modest increase of ROS generating phagocytes, this increased ROS production in the *Muc2*^−/−^ intestine may be part of the adaptive stress/repair response to increased damage/challenge due to the altered interaction between the IECs and the environment (eg. the gut bacteria) to control tissue homeostasis. However, when this stress response persists, it results in chronic, low level inflammation, also termed para-inflammation, that can drive tumorigenesis [30]. It is of note that these data are also consistent with the phenotype of double mutant *Muc2*^−/−^; *Apc*^1638/N^ mice in which we demonstrated that there was a shift of tumor burden towards the colon, where we have also documented increased ROS producing phagocyte infiltration (Supplementary Figure 3); the phenotype of the double mutant *Muc2/Apc* mouse is reminiscent of the shift in tumor location observed in the *Apc* mutant mice challenged to mount of an inflammatory response. In addition, a large proportion of tumors in *Muc2*^−/−^*Apc*^1638/N^ mice were characterized by mutational inactivation of the wild type *Apc* allele, in contrast to the loss of heterozygosity which is the most common mechanism of inactivation of the WT *Apc* allele in tumors of *Apc*^1638/N^ mice in which the mucus barrier is intact [5].

We have documented alterations of lipid metabolism pathways in IECs of the normal appearing mucosa of the *Muc2*^−/−^ mouse. In some instances these changes (i.e. Acot1 and Acly1) are modest though statistically significant; it can be inferred that they translate into shifted metabolic pathways consistent with the increased proliferation in *Muc2* mice. Interestingly similar alterations involving lipid adsorption, processing and chylomicron release have been documented in the intestinal epithelium of mice with enterocyte specific inactivation of the retinoblastoma protein (RB) [31], resulting in enhanced crypt cell proliferation. In the absence of clear pathology, the alterations of lipid metabolism may be linked to an expansion of the proliferative capacity of IECs in response to damage signals, as in the case of low level chronic inflammation in *Muc2^−/−^* mice, or intrinsic signals in response to alterations of the Rb pathway. Interestingly, data generated using individual metabolic chambers to measure food intake, locomotor activity, oxygen consumption, and body temperature, accurately and with high temporal resolution, in several individual WT and *Muc2^−/−^* mice, showed that the altered expression of lipid metabolism genes was not accompanied by variations in body composition or rate of metabolism (data not shown). These results are similar to the reported absence of variations in body composition and rate of metabolism in comparing Rb deficient and WT mice. In this context, it is notable that alterations of fatty acid metabolism are documented in IBD [32]. Thus, in response to chronic damage intestinal epithelial cells may switch to a metabolic state characterized by altered lipid and enhanced glycolytic metabolism associated with increased proliferation. In agreement, analysis of the expression profile of IECs along the crypt-villus axis in wild type Black6 mice [33] shows that the decreased expression of genes involved in lipid metabolism that characterize the IECs of *Muc2*^−/−^ mice recapitulates the normal developmental pattern of expression along the crypt/villus axis (Table 2), consistent with the hypothesis that decreased lipid metabolism characterizes cells of the crypt proliferative compartment.

Finally, alterations of genes involved in lipid metabolism, specifically of *Mttp*, may also affect mucosal immunity. MTTP, in cooperation with IEC-expressed CD1d, provides protective functions in inflammation through induction of IL10, Hsp105 and Cd11d to itself [34]. Thus, reduced Mttp expression may perpetuate the cycle of a chronic “wound-healing” response to low levels of tissue damage that is associated with increased risk of tumor development [30].

The presence of a chronic stress response well before the appearance of tumors in *Muc2*^−/−^ mice can also be inferred by the altered expression of components of the ER-stress response. In addition, the increased accumulation of Xbp1-s, which was shown to be required for the maintenance of secretory cell lineages [35], may also be linked to the ER response associated with enhanced production of bacteriostatic and bacteriocidal peptides by secretory Paneth cells that we and others previously reported [5, 24, 25], documented by their increased expression of Grp78 (Figure 9A and 9B). Of note, conditional inactivation of *Xbp1* in the intestine results in spontaneous colitis, and, in IBD patients, increased levels of *Xbp1-s* were described [35, 36]. Studies in several mouse models with alterations of components of the UPR specifically in intestinal goblet cells, or displaying an exacerbated ER response linked to a mutation in the *Muc2* gene resulting in the accumulation of miss-folded protein, have underlined the complex interplay between goblet cells and the Muc2 mucin they secrete, and the ER-response in determining increased susceptibility to colitis [37–40]. However, we did not detect an enhanced ER stress response specifically in the goblet cells of the *Muc2*^−/−^ mouse. In this context, it is important that the ER stress response in *Muc2*^−/−^ intestine is not accompanied by colitis, but may be a marker of the lower level of chronic inflammation that we have previously reported in the *Muc2*^−/−^ mucosa. This status of pathologically silent chronic inflammation may explain the increased Grp78 positivity of intravillus stroma cells that may be in an activated state with increased expression/secretion of cytokines, as previously reported by us [2]. Similar observation was reported in the small intestine of IEC-specific *Grp78-deficient* mouse [41].

The significance of altered cross-talk between the intestinal epithelial cells and the microbiota in *Muc2*^−/−^ mice is at least two fold. First, more subtle and focal compromise of the mucus barrier, indicated by a decrease in goblet cells, the lineage that synthesizes and secretes mucins and other components of the mucus, is seen in many mouse models of intestinal cancer [42–45]. Moreover, aberrant crypt foci, and a subset of mucin depleted ACF (MDF) which may be direct precursors to tumor development [44, 46–48] are depleted in goblet cells and thus, focally in the mucins they produce. Thus, these focal changes may mimic the generalized defect of mucosal barrier function generated in the *Muc2* mouse that leads to development of tumors in the small and large intestine and the rectum. Interestingly, even in sporadic colon cancers, which are not clearly associated with pre-existing inflammation, Grivennikov et al. identified an inflammatory signature linked to an adenoma-associated barrier defect and microbial product exposure [49], involving IL23, which we reported was elevated in the normal appearing colon of *Muc2*^−/−^ mice [5].

In summary, our analysis of the *Muc2*^−/−^ mouse, in which there is loss of the mucus barrier, reveals important perturbations that contribute to the loss of homeostasis and the development of colon and intestinal tumors. These perturbations, which are amplified by their presence throughout the mucosa in the *Muc2* mouse completely devoid of the mucus barrier, may identify changes in biochemistry and molecular pathways that drive higher probability of tumor development but which are only subtly and focally altered in the long-term development of common sporadic colon cancer.

## MATERIALS AND METHODS

### Mice

The *Muc2*^−/−^ mice have been back-crossed onto a Bl/6 background for >10 generations. *Muc2*^−/−^ and *Muc2*^+/+^ littermates from breeding of heterozygotes were maintained on standard chow diet and provided tap water *ad libitum*. All mice were housed and bred in an AALAC accredited animal services facility and all mouse experiments followed a protocol approved by the Institutional Animal Care and Use Committees at the Albert Einstein School of Medicine. Mice were sacrificed at 3 month of age.

### Total RNA isolation and microarray analysis

Total RNA was isolated from frozen, pulverized tissue, or purified intestinal epithelial cells, using Trizol (Invitrogen), treated with RQ1 RNAse free DNAseI (Promega) for 15 min at room temperature followed by purification on a Qiagen column (RNeasy kit, Qiagen) following the kit protocol for those samples to be used in microarray experiments. Otherwise, after DNAse I digestion, RNA was purified by sequential phenol:chloroform extraction followed by ethanol precipitation. Integrity of RNA was determined using the Agilent 2100 Bioanalyzer or by ethidium bromide visualization upon agarose gel electrophoresis.

For microarray experiments, total RNA was purified from intestinal epithelial cells isolated from the duodenum of age matched *Muc2*^−/−^ and WT mice, three mice per genotype, at 3 months of age, both genders in equal number, as previously reported [5]. Duodenum was chosen as it was the preferential site for tumor development [4]. Expression analysis was performed using Gene Chip Mouse Genome 430 2.0 arrays (Affimetrix) by the Albert Einstein core facility (http://www.aecom.yu.edu/dna/affy). Background correction, normalization, and log-scale transformation of the raw expression data was done by robust multichip analysis (RMA) using the Methods for Affymetrix Oligonucleotide Arrays R package (freely available at http://www.bioconductor.org). The criteria to consider a gene to be altered in expression in the flat mucosa of *Muc2*^−/−^ mice compared to wt mice were a mean difference in expression level (increase or decrease) of 1.5 fold, and a *p*-value comparing *Muc2*^−/−^ to WT mice of < 0.05 (Student's *t*-test).

Genes satisfying these criteria were further analyzed using IPA to identify biological functions and canonical pathways affected by Muc2 deficiency.

### Quantitative RT-PCR

Microarray validation of selected genes was performed by reverse transcription of RNA preparations from duodenal IECs purified independently from a separate cohort of 3 month old mice (3 mice/genotype). Expression levels of individual genes were determined by qRT-PCR using SYBER Green Core reagent kit in a 7900HT ABI Instrument (Applied Biosystem), using the standard curve method with values normalized to those of the ribosomal protein L41 (*Rpl41*).

Total RNA was isolated from independently purified intestinal epithelial cells from duodenum from 3 *Muc2^−/−^* and 3 WT, 3-month-old, gender matched mice. Cells were rapidly frozen in liquid N2, and kept at −80°C till RNA isolation. RNA was reverse transcribed into cDNA using SuperScript III reverse transcriptase (Invitrogen) and anchored oligodT primers. Amplification was conducted in a 7900HT ABI instrumente (Applied Biosystems) using the SYBER Green Core reagent kit. Each sample was analyzed in duplicate and quantification of relative level of gene expression was determined using the standard curve method. All values were normalized to the levels of β-actin or ribosomal protein L41 (*Rpl41*). In all cases reaction specificity was evaluated by the analysis of product melting curves and gel analysis. Primer sequences are available upon request

### Immunohistochemistry

5 μm sections from formalin fixed, paraffin embedded samples were processed as previously reported [50–52]. Briefly, after deparaffinization and rehydration, sections were quenched in 3% H_2_O_2_ for 10 minutes at room temperature, briefly washed in dH_2_O. Antigen retrieval was then routinely performed in 0.01M sodium citrate pH 6.0 for 30′ in a conventional rice steamer, and slides then allowed to cool on the bench for 30 minutes. Blocking, when required, was performed by incubating slides in blocking solution [2.5% non-fat dried milk, 2% BSA fraction V, (Sigma) in TBS-T (1X TBS + 0.05% tween-20)] for 45 minutes at room temperature. Primary antibody, diluted in Signal Stain antibody diluent (Cell Signaling), was applied overnight at 4°C, followed by the SupperPicture anti rabbit HRP-conjugated polymer 1h at RT (*In Vitro*gen).). Detection was achieved using 3′5′ diaminobenzidine (DAB) as substrate. Antibodies were: anti g-H2AX/Phospho-Histone H2AX (Ser 139), rabbit monoclonal – Cell Signaling (1:400). Anti Grp78/BiP, rabbit monoclonal – Cell Signalling (1:200). Anti ITF/TFF3, rabbit polyclonal, was a generous gift of Dr. Tomasetto, Strasburg, France, (1:500).

## SUPPLEMENTARY FIGURES


