# Supplementary material for: MUC2 mucin deficiency alters inflammatory and metabolic pathways in the mouse intestinal mucosa
Source: Oncotarget. 2017 Apr 6;8(42):71456–70. doi: 10.18632/oncotarget.16886 (PMC5641062; doi:10.18632/oncotarget.16886)
Supplement: Supplementary file 1 [file oncotarget-08-71456-s001.pdf]

MUC2 mucin deficiency alters inflammatory and metabolic pathways in the mouse intestinal mucosa

Supplementary Materials

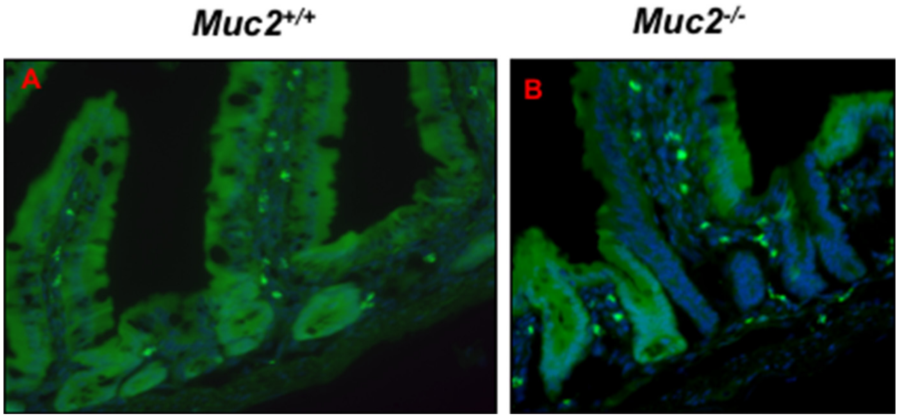

Supplementary Figure 1: ROS producing cell detection in WT and *Muc2*$^{-/-}$ mice. Tissue was probed by immunofluorescent detection of oxidized DCF. Magnification 400 ×

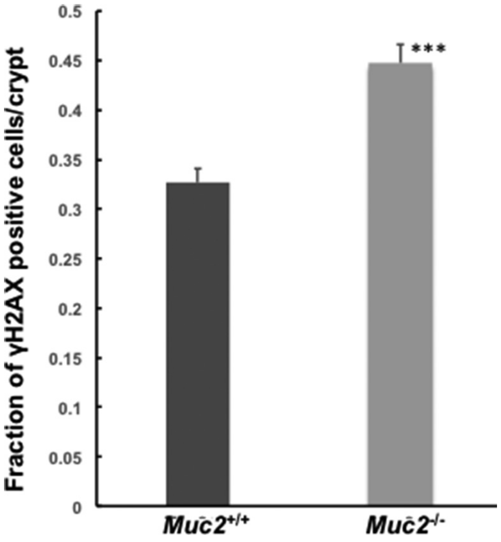

Supplementary Figure 2: Increased number of γH2AX positive cells in *Muc2*$^{-/-}$ crypts. Quantification of γH2AX-positive cells in sections from *Muc2*$^{+/+}$ and *Muc*$^{2-/-}$ mice. The number of γH2AX-positive cells was determined in tissue sections incubated with anti γH2AX-antibodies, as shown in Figure 2.

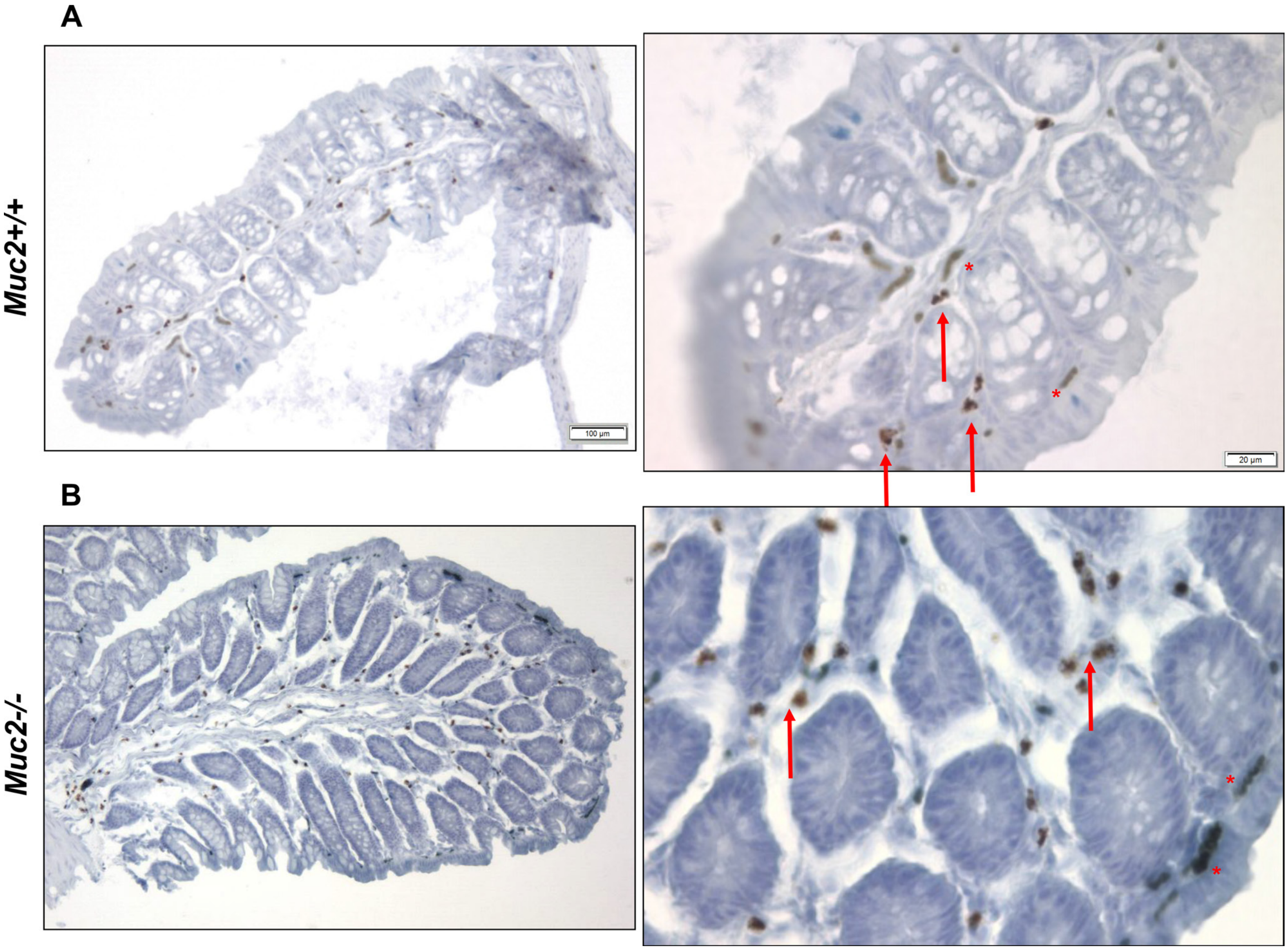

Supplementary Figure 3: Increased number of ROS positive cells in the colon of *Muc2*$^{-/-}$ mice. Representative micrographs of frozen sections of flat mucosa of colon from 3 month old *Muc2*$^{+/+}$(**A**) and *Muc2*$^{-/-}$ (**B**) mice, respectively, incubated *in vitro* with DAB, as described in Material and Methods. Red arrows indicate cell positivity due to DAB polymerization in the presence of cell-generated H_2O_2. Red asterisks indicate red blood cells.
